# Mortality Predicted Accuracy for Hepatocellular Carcinoma Patients with Hepatic Resection Using Artificial Neural Network

**DOI:** 10.1155/2013/201976

**Published:** 2013-04-30

**Authors:** Herng-Chia Chiu, Te-Wei Ho, King-Teh Lee, Hong-Yaw Chen, Wen-Hsien Ho

**Affiliations:** ^1^Department of Healthcare Administration and Medical Informatics, Kaohsiung Medical University, 100 Shi-Chuan 1st Road, Kaohsiung 807, Taiwan; ^2^Bureau of Health Promotion, Department of Health, No. 2 Changqing St., Xinzhuang, New Taipei City 242, Taiwan; ^3^Department of Surgery, Kaohsiung Medical University Hospital, 100 Shi-Chuan 1st Road, Kaoshiung 807, Kaohsiung, Taiwan; ^4^Yuan's Hospital, No. 162 Cheng Kung 1st Road, Kaohsiung 802, Kaohsiung, Taiwan

## Abstract

The aim of this present study is firstly to compare significant predictors of mortality for hepatocellular carcinoma (HCC) patients undergoing resection between artificial neural network (ANN) and logistic regression (LR) models and secondly to evaluate the predictive accuracy of ANN and LR in different survival year estimation models. We constructed a prognostic model for 434 patients with 21 potential input variables by Cox regression model. Model performance was measured by numbers of significant predictors and predictive accuracy. The results indicated that ANN had double to triple numbers of significant predictors at 1-, 3-, and 5-year survival models as compared with LR models. Scores of accuracy, sensitivity, specificity, and area under the receiver operating characteristic curve (AUROC) of 1-, 3-, and 5-year survival estimation models using ANN were superior to those of LR in all the training sets and most of the validation sets. The study demonstrated that ANN not only had a great number of predictors of mortality variables but also provided accurate prediction, as compared with conventional methods. It is suggested that physicians consider using data mining methods as supplemental tools for clinical decision-making and prognostic evaluation.

## 1. Introduction

Hepatocellular carcinoma (HCC) is the fifth common cancer and the third leading cause of death worldwide. According to the World Health Organization (WHO) statistics in 2000, it has been estimated that there are at least 564,000 new cases of HCC per year around the world [1]. Though Asia and Africa have accounted for 80% of incidence cases of HCC for years, the incidence rates have been found to be significantly increasing in the United States [2] and some European nations [3]. 

Hepatic resection is one of the most effective treatments and the standard modality to achieve a long-term survival for HCC [4, 5]. However, even with progress in diagnosis and treatment, the overall mortality in HCC patients is still higher than in other types of cancer patients. The factors associated with mortality have been explored by traditional statistical methods, such as logistic regression (LR) and Cox regression [6]. Logistic analysis models hypothesize that as mean values of a given predictor variable increase, the predicted risk of the outcome increases. Despite its recognized limitations [7], LR is still widely used in clinical outcome studies.

Recently, artificial neural networks (ANNs) have proven effective for nonlinear mapping based on human knowledge [8]. Like a network of brain neurons, an ANN containing multiple layers of simple computing nodes can accurately approximate continuous nonlinear functions and can reveal previously unknown relationships between given input and output variables [8–10]. The unique structure of ANNs is well suited for machine learning methods such as backpropagation [11] and evolutionary algorithms [8, 12, 13]. Because of their universal approximation capability, potential applications of ANNs have attracted interest in some fields [14–18]. The novel application of ANN in this study was in predicting postresection prognosis in HCC patients in order to enhance their clinical management by quantifying expected risks.

To our knowledge, no study has applied ANN in predicting the prognosis of HCC patients after resection. Additionally, despite the numerous comparisons of ANN and LR in the literature, no study has convincingly demonstrated which is superior in terms of predictive accuracy [19]. The objectives of the study are accordingly, firstly, to construct an ANN model and predict the input variables associated with the mortality of HCC patients undergoing resection and examine the differences in significant predictors between the ANN and LR models, and secondly, to compare the predictive accuracy of ANN and LR in different survival year estimation models.

## 2. Patients and Methods

The inclusion and characteristics of the study population are the same as those described in the previous report [6]. Briefly, the study population consisted of 608 consecutive patients with HCC who underwent liver resection at Kaohsiung Medical University Hospital and Yuan's Hospital in Taiwan. In this study, we first excluded patients who received or underwent the following treatments or conditions: (i) received liver resection before (*n* = 20); (ii) treatments with radiofrequency ablation (*n* = 24) and microwave ablation (*n* = 15); (iii) histopathological reports indicated benign tumor and/or nonprimary liver cancer (*n* = 27); (iv) had case history missing and/or was incomplete (*n* = 34); (v) expired within thirty days after surgery (*n* = 5); and (vi) tumor remained after resection (*n* = 1). Further, to enhance data completeness, we excluded patients with missing values in key explained variables (*n* = 30) and patient follow-up days of less than one year (*n* = 18). Finally, 434, 341, and 264 were included in 1-, 3-, and 5-year survival groups, respectively. 

There were two sources of data examined and used in our study: patient clinical information and death registry data. Patients' clinical information was derived from medical charts and review by attending physician from both hospitals using a constructed questionnaire. The information included patients' demographics and hepatic biochemical parameters. The mortality data bank is established and maintained by the Statistics Office, Department of Health, Taiwan. Two datasets were merged by unique identifier. All patients were followed until death or December 31, 2008, whichever came first.

### 2.1. Development of the Artificial Neural Network Models

Waikato Environment for Knowledge Analysis (WEKA) software V3.6.0 (with backpropagation algorithm) was used to construct the ANN model. This user-friendly software is compatible with Microsoft Windows and has been validated for use in developing new machine learning schemes [20]. 

The outcome variables in this study were death during the study period (event) and survival (no event), which were coded as 1 and 0, respectively. To minimize the effects of extreme values and to enhance the computing efficiency of the ANN model, all continuous explanatory variables were first transformed into categorical variables. The cut-off points for these variables were based on those used in previous clinical studies [6, 21–25]. Low and high risk were coded as 0 and 1, respectively. The variables included BUN AST, *α*-fetoprotein, ALT, total bilirubin, and others. Other recoded items included TNM stage, a common prognostic index of cancer risk or severity, and ASA, a risk score for surgical procedures, were also recoded. The TNM stage ranges from 1 to 6, and ASA score ranges from 1 to 4. Two variables were recoded as 0 for low risk, 1 for medium risk, and 2 for high risk (Table 1). High risk was assumed to increase the probability of death (event).

Model development in this study was performed in two stages. Firstly, to enhance the calculation efficiency and prediction performance of the ANN model construct, a univariate Cox proportional hazard model was used to test variables for potential associations with survival or death. Variables with statistically significant (log-rank test) associations with survival were retained to construct the ANN model (Table 1). Of the 33 input variables, the following 21 statistically significant variables were retained for constructing ANN models: age, comorbidity, liver cirrhosis, *α*-Fetoprotein, AST, total bilirubin, albumin, BUN, platelet, ASA classification, Child-Pugh classification, TNM stage, tumor number, tumor size, portal vein invasion, biliary invasion, surgical procedure, postoperative complication, recurrence, and postoperative treatment. Additionally, gender was included as a control variable. 

**Figure 1:**
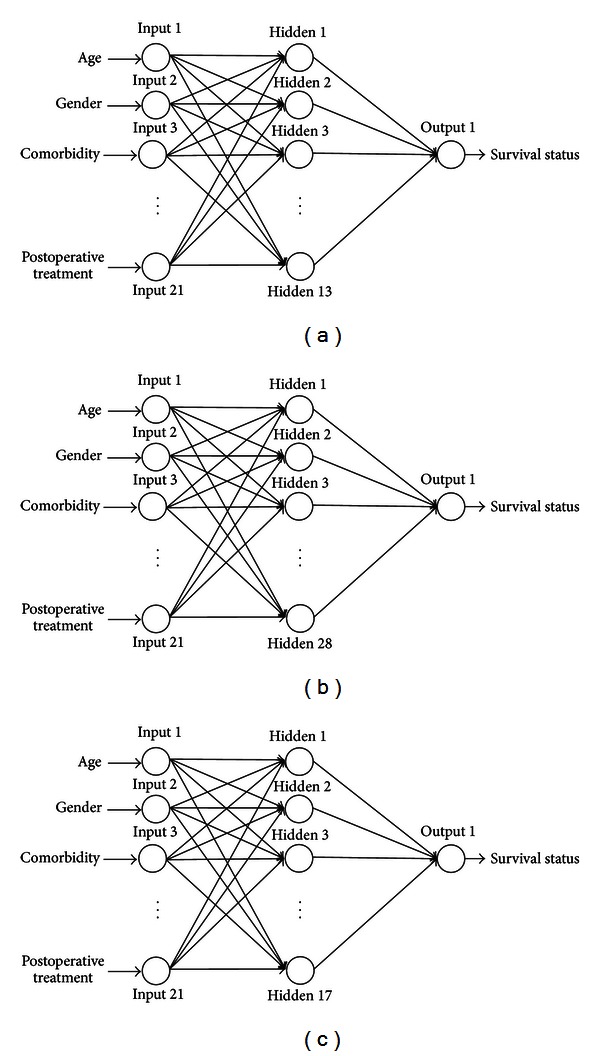
(a) Artificial neural network model for 1-year survival. (b) Artificial neural network model for 3-year survival. (c) Artificial neural network model for 5-year survival.

Secondly, Figure 1 shows the numbers of neurons in the input, hidden, and output layers of the ANN models of 1-,   3-, and 5-year survival. In all three models, the input layers contained 21 neurons. In the hidden layers, the numbers of neurons were optimized using training and validation data in a trial-and-error process to maximize predictive accuracy [26], which resulted in 13, 28, and 17 neurons in the 1-, 3-, and 5-year models, respectively. The output layer in all models contained only one neuron, which represented survival status.

Studies suggest that an ROC plot should present the trade-off between sensitivity and specificity for all possible cut-offs [27]. The SPSS Windows version 6.1 software used for model building in this study automatically generated 110 possible cut-offs for each of the 1-, 3-, and 5-year models. For each of the three models, the authors then selected the best cut-off in terms of accuracy, sensitivity, and specificity.

### 2.2. Training Groups and Validation Groups

The 1-, 3-, and 5-year survival data were randomly divided into training sets and validation sets. The training data set was used to develop the model whereas the validation data set was used to assess its predictive accuracy [28]. In accordance with the literature, 80% of the data were used for training, and the remaining 20% were used for validation [29, 30]. In the 1-year survival group, for example, data for 347 and 87 patients were used for training and for validation, respectively. Data validation is needed to avoid overtraining an ANN to recognize specific subjects in the training data rather than learning general predictive values. Additionally, *χ* ^2^ and Fisher's exact test analysis were performed to compare the effects of each input variable in terms of training and validation.  Table 2 shows that the effects of all input variables in all three survival models did not significantly differ between training and validation, which confirmed the reliability of the data selection.

In accordance with the criteria used for performance comparisons reported in the literature, the ANN and LR models were compared in terms of overall accuracy (sum of correct predictions divided by total predictions), sensitivity, specificity, and area under the receiver operating characteristic curve (AUROC) [9, 14]. Higher scores were considered better for validation. In the WEKA program, ANN model parameters for learning rate, momentum, and training time were set to 0.3, 0.2, and 500, respectively.

## 3. Results

In this section, the significant predictors were selected according to predictive error ratio (greater than one) for 1-, 3-, and 5-year survival models using ANN and LR in the order of features of demographic, clinical, surgical outcome, and prognosis. Overall, ANN models had more significant input variables at 1-,   3-, and 5-year survival models than that of LR models. More specially, ANN had 15, 13, and 9 significant predictors at 1-, 3-, and 5-year survival models, whereas LR only had 8, 4, and 4 variables accordingly. 

Notably, six variables in the clinical features dimension were significant predictors in all three survival models constructed by ANN: comorbidity, liver cirrhosis, *α*-Fetoprotein, platelet, ASA classification, and TNM stage. Among these variables, liver cirrhosis, *α*-Fetoprotein, and TNM stage were significant predictors for the LR model at 1-year survival model but were consistently significant for ANN at 1-, 3-, and 5-year models. 

Table 4 shows the accuracy, sensitivity, and specificity of the 1-, 3-, and 5-year survival estimation models using ANN and LR of the training groups. All three performance criteria were superior in the models using ANN to those using LR in any survival estimation models. For the 1-year survival ANN model, the accuracy was 99.1% in contrast with the 1-year survival model using LR, whose accuracy was 89.0%. Sensitivity for ANN was 100% at the 5-year survival model compared to 67.5% for LR. Specificity for ANN was 96.2% at the 1-year model whereas it was 34.6% for LR. 

Table 5 shows the accuracy, sensitivity, and specificity of the 1-, 3-, and 5-year survival estimation models using ANN and LR for validation groups. Although the results were mixed in scores of accuracy, sensitivity, and specificity between ANN and LR, most performance criteria were superior in the models by using ANN to those using LR in any survival models. Take the 5-year survival model, for example, the accuracy was 79.2% for ANN, whereas LR was 70.6%. LR had a relatively higher score (94.9%) in specificity measure at 1-year survival model, but poor value in specificity (25.0%). In contrast, ANN had relatively higher values at both scores in sensitivity (88.6%) and specificity (50.0%).

**Figure 2:**
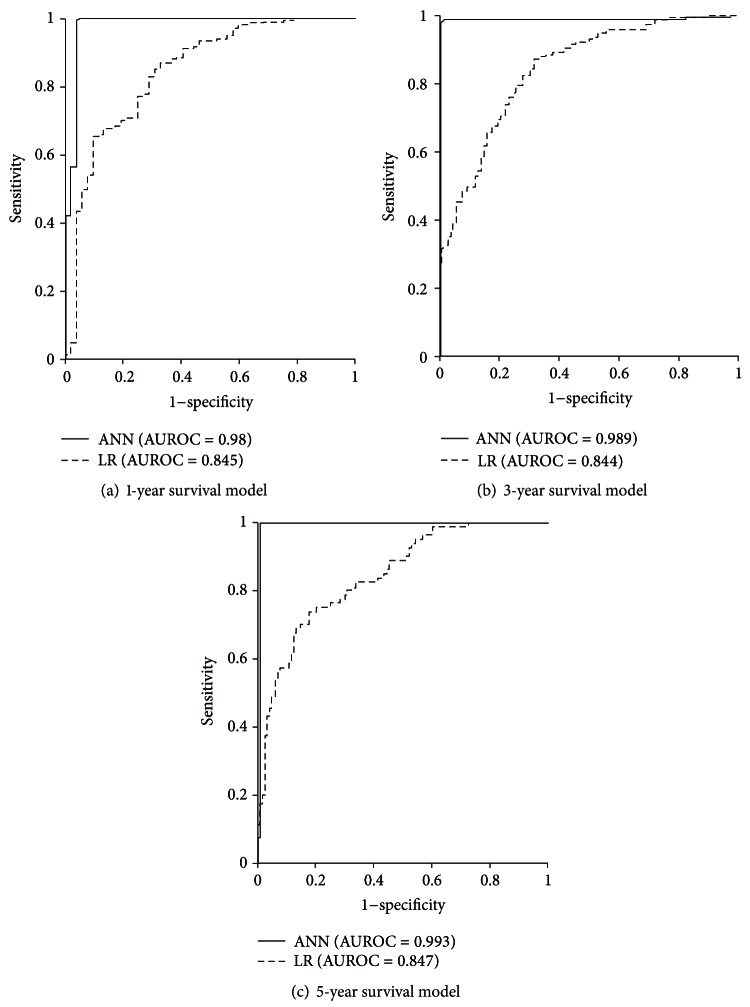
ROC curves and AUROCs for ANN and LR models of 1-, 3-, and 5-year survival when using training data.

**Figure 3:**
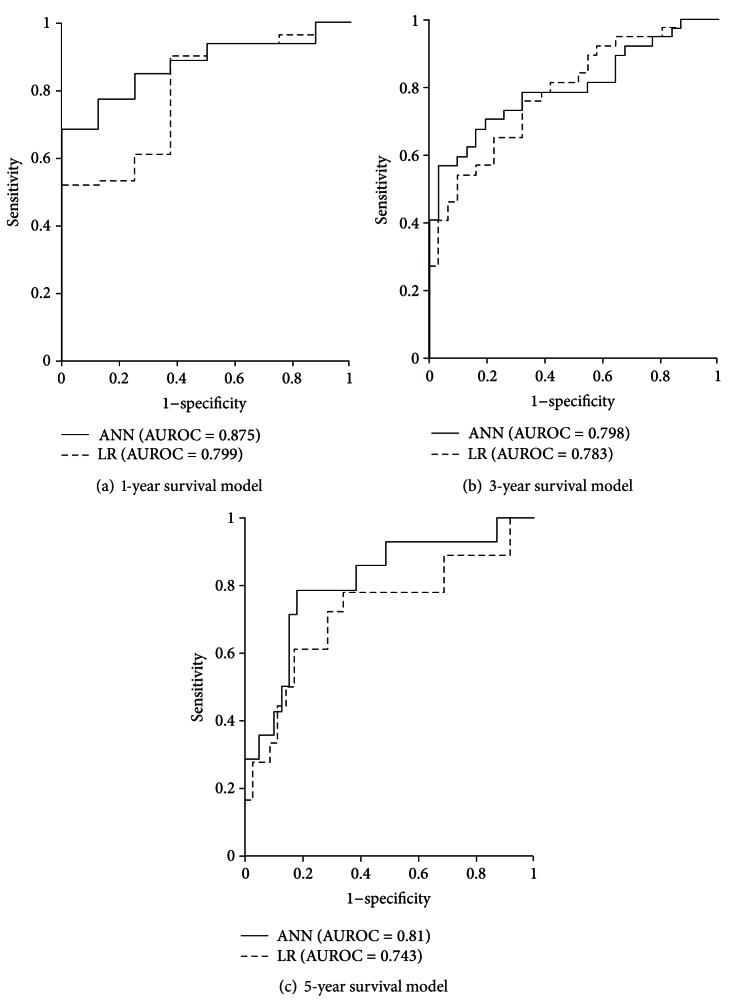
ROC curves and AUROCs for ANN and LR models of 1-, 3-, and 5-year survival when using validation data.

AUROCs for training data and validation data (Figures 2 and 3, resp.) were significantly higher in ANN models than in LR models. For training data, 1-, 3-, and 5-year survival AUROCs were 0.980, 0.989, and 0.993 in ANN models and 0.845, 0.844, and 0.847 in LR models, respectively. For validation data, the 1-, 3-, and 5-year survival AUROCs were 0.875, 0.798, and 0.810 in ANN models and 0.799, 0.783, and 0.743 in LR models, respectively.

## 4. Discussion

We have created models for prediction of outcome of HCC patients undergoing resection using ANN with input variables which were found to be significantly associated at univariate analysis. Clinical factors such as comorbidity, liver cirrhosis, *α*-Fetoprotein, platelet, ASA classification, and TNM stage were significant for 1-, 3-, and 5-year survival in ANN models as shown in Table 3. Among those, only liver cirrhosis, *α*-fetoprotein, and TNM stage were also found significant for LR at the 1-year prediction model. The consistently significant variables in mortality are suggested to be reviewed by clinicians to examine both short- and long-term clinical outcomes for HCC patients. 

The appropriate selection of input variables is vital to the success of ANN construction. The process improves efficiency of the ANN model's appropriate complexity (by using the most predictive variables) and low redundancy. We first employed traditional statistics to select those variables statistically significant as input variables to make equal comparative analysis. The crude hazard ratio has been widely used by biostatisticians and clinicians to explore the difference between crude and adjusted hazard ratio.

Our study found that ANN had double to triple numbers of significant predictors at 1-, 3-, and 5-year survival models as compared with LR models. A previous study also found such a gap between models derived from ANN and traditional statistical methods [17]. The reason for the difference might be owing to the fact that models derived from logistic regression usually employ variables that are statistically significant predictors of the outcome, and ANN utilizes all possible interactions between all input variables and the outcome, regardless of their statistical significance. ANN can be developed using a number of different training algorithms, many of which are continually being developed and may offer improved prediction accuracy. On the other hand, ANN cannot provide detailed information such as the hazard ratio, which generally provides direction and magnitude of individual variables on outcome variables. 

As compared with the 1-year mortality model, numbers of predictors at both ANN and LR models decreased at 3- and 5-year survival models, though the ANN model appeared to have lower decreased rates. This suggested that relationship between input variables and survival status may be correlated rather than simply for the prediction of short-term outcome, and that 3- and 5-year survival status may be confounded by factors that are more complex. The change in health status over time should be examined to have better knowledge on long-term survival estimation. 

In all training sets and in most validation sets, accuracy, sensitivity, specificity, and AUROC were higher in the 1-, 3-, and 5-year survival models constructed by ANN than in those constructed by LR, which is consistent with other reports that ANN outperforms LR in both training [15, 31–35] and validation [14, 36, 37].

Although the ANN models in the current study generally had higher sensitivity and specificity compared to LR models when using both training data and validation data, a notable exception was specificity when using validation data in the 1-year LR model (Table 5). Compared to the 1-year ANN model, the 1-year LR model had higher sensitivity (94.9%), higher accuracy (88.5%) but lower specificity (25.0%) when using validation data. The literature [38] suggests that specificity and sensitivity values lower than 40% should be considered poor. Sensitivity and specificity are important when testing the capability of a model to recognize positive and negative outcomes. Sensitivity and specificity must also be measured to determine the proportion of false negatives or false positives produced by a model [39]. Comparing false positive and false negative rates explains the tendency of a model to misclassify positive patients as negative patients and vice versa [40]. Ideally, both sensitivity and specificity should be high [40]. According to comparisons of ANN and LR models reported in the literature as well as the experimental results in this study, ANN models have fewer prediction errors.

Although the proposed ANN-based models generally outperformed LR models in this study, the findings of this study should be interpreted cautiously. First, the WEKA program cannot be used if the ANN is constructed with numerous input variables, which can cause “insufficient computer memory” error messages. However, the number of input variables used in the present study was 21 suitable for the program used. Second, an ROC plot should be constructed for all possible cut-offs for a clear representation of the trade-off between specificity and sensitivity. Since the cut-offs used for each of the 1-, 3-, and 5-year survival models in this study were selected by the authors from possible cut-offs generated by a statistical software package, bias could not be ruled out. Third, although previous works adopted a 20% validation group [29, 30], this study adopted 25% and 30% validation groups to detect the sample difference. Therefore, the potential treat from the sample should be noted. Fourth, since the HCC patient sample in the current study was derived from only two hospitals, the ability to generalize the findings is limited. For a stronger methodological conclusion, future studies should test external validity such as by analyzing hepatic resection outcomes in HCC patients treated in different medical institutions.

## 5. Conclusions

In conclusion, survival estimation models at 1-, 3-, and 5-year intervals for HCC patients undergoing hepatic resection could be constructed by ANN, a data mining method as compared with conventional logistic regression. Arguably more significant predictors of mortality were identified by ANN at 1-, 3-, and 5-year models as compared with LR. The values in accuracy, sensitivity, specificity, and AUROC of ANN models were generally higher than those of LR models.

The study supported previous studies that ANN had better performance in prediction as compared with LR. The study suggested that ANN could become one tool for predicting clinical short- and long-term outcomes. It is suggested that physicians consider using data mining methods as a supplemental tool to make clinical decision-making and prognostic evaluation.

## Figures and Tables

**Table 1:** Potential input variables and output variable for prognostic models.

Variables	Value	*P* value
Input variable:		
Demographic		
Age (years)*	0: ≦65, 1: >65 (mean = 57.7)	0.04
Gender	0: male, 1: female	0.37
Clinical features		
Comorbidity*	0: no, 1: yes	0.04
Liver cirrhosis*	0: no, 1: yes	<0.001
Chronic hepatitis	0: no, 1: HBV, 2: HCV, 3: HBCV	0.68, 0.12, 0.48
*α*-Fetoprotein (ng/mL)*	0: ≦100, 1: >100	<0.001
AST (U/L)*	0: ≦80, 1: >80	<0.001
ALT (U/L)	0: ≦80, 1: >80	0.07
Total bilirubin (mg/dL)*	0: ≦1.0, 1: >1.0	0.01
Albumin (g/dL)*	0: >3.5, 1: ≦3.5	<0.001
BUN (mg/dL)*	0: ≦21, 1: >21	0.01
Creatinine (mg/dL)	0: ≦1.4, 1: >1.4	0.24
Platelet (10^3^/*μ*L)*	0: >150, 1: ≦150	0.02
Prothrombin time (%)	0: ≦80, 1: >80	0.43
ICGR_15_ (%)	0: ≦15, 1: >15	0.30
ASA classification*	0: ASA = 1, 1: ASA = 2, 2: ASA = 3	0.01, 0.94
Child-Pugh classification*	0: A, 1: B, C	<0.001
TNM Stage*	0: I, 1: II, 2: IIIa, IIIb, IIIc, IV	<0.001, <0.001
Tumor number*	0: single, 1: multiple	<0.001
Tumor size (cm)*	0: ≦5, 1: >5	<0.001
Portal vein invasion*	0: no, 1: yes	<0.001
Biliary invasion*	0: no, 1: yes	0.01
Surgical process and outcome		
Surgical procedure*	0: laparoscopic, 1: open surgery	0.02
Extent of resection	0: minor, 1: major	0.12
Resection margin (mm)	0: >10, 1: ≦10	0.08
Surgical time (minutes)	0: ≦180, 1: >180	0.75
Blood loss (mL)	0: ≦1000, 1: >1000	0.29
Blood transfusion	0: no, 1: yes	0.55
Blood transfusion (mL)	0: ≦1000, 1: >1000	0.07
Postoperative complication*	0: no, 1: yes	<0.001
Prognostic		
Recurrence*	0: no, 1: yes	<0.001
Preoperative treatment	0: no, 1: yes	0.08
Postoperative treatment*	0: no, 1: yes	<0.001
Output variable:		
Status	0: survival, 1: dead	

*Significant input variables.

**Table 2:** Comparison of clinical features between training data and validation data.

Variables	Definitions	1-year (*N* = 434)					3-year (*N* = 341)					5-year (*N* = 264)				
		Training		Validation			Training		Validation			Training		Validation		
		(*N* = 347)		(*N* = 87)		*P*	(*N* = 273)		(*N* = 68)		*P*	(*N* = 211)		(*N* = 53)		*P*
		*N*	%	*N*	%		*N*	%	*N*	%		*N*	%	*N*	%	
Age	≦65	251	72.3	62	71.3	0.842	197	72.2	53	77.9	0.335	157	74.4	37	69.8	0.498
	>65	96	27.7	25	28.7		76	27.8	15	22.1		54	25.6	16	30.2	
Gender	Male	262	75.5	68	78.2	0.604	209	76.6	54	79.4	0.616	162	76.8	38	71.7	0.440
	Female	85	24.5	19	21.8		64	23.4	14	20.6		49	23.2	15	28.3	
Comorbidity	No	170	49.0	39	44.8	0.487	144	52.7	35	51.5	0.850	112	53.1	31	58.5	0.480
	Yes	177	51.0	48	55.2		129	47.3	33	48.5		99	46.9	22	41.5	
Liver cirrhosis	No	118	34.0	30	34.5	0.933	91	33.3	19	27.9	0.395	63	29.9	11	20.8	0.187
	Yes	229	66.0	57	65.5		182	66.7	49	72.1		148	70.1	42	79.2	
*α*-Fetoprotein (ng/mL)	≦100	243	70.0	62	71.3	0.822	192	70.3	43	63.2	0.258	135	64.0	38	71.7	0.291
	>100	104	30.0	25	28.7		81	29.7	25	36.8		76	36.0	15	28.3	
AST	≦80	283	81.6	72	82.8	0.795	222	81.3	53	77.9	0.528	169	80.1	40	75.5	0.459
	>80	64	18.4	15	17.2		51	18.7	15	22.1		42	19.9	13	24.5	
Total bilirubin	≦1.0	255	73.5	57	65.5	0.139	200	73.3	52	76.5	0.590	152	72.0	41	77.4	0.435
	>1.0	92	26.5	30	34.5		73	26.7	16	23.5		59	28.0	12	22.6	
Albumin	>3.5	270	77.8	68	78.2	0.944	210	76.9	52	76.5	0.937	153	72.5	39	73.6	0.875
	≦3.5	77	22.2	19	21.8		63	23.1	16	23.5		58	27.5	14	26.4	
BUN	≦21	293	84.4	75	86.2	0.681	231	84.6	57	83.8	0.872	174	82.5	46	86.8	0.450
	>21	54	15.6	12	13.8		42	15.4	11	16.2		37	17.5	7	13.2	
Platelet	>150	170	49.0	48	55.2	0.303	133	48.7	36	52.9	0.533	99	46.9	23	43.4	0.646
	≦150	177	51.0	39	44.8		140	51.3	32	47.1		112	53.1	30	56.6	
ASA classification	1	87	25.1	19	21.8	0.616	75	27.5	21	30.9	0.599	63	29.9	23	43.4	0.149
	2	179	51.6	50	57.5		147	53.8	32	47.1		106	50.2	23	43.4	
	3, 4	81	23.3	18	20.7		51	18.7	15	22.1		42	19.9	7	13.2	
Child-Pugh classification	A	335	96.5	85	97.7	0.584	263	96.3	67	98.5	0.360	201	95.3	52	98.1	0.353
	B, C	12	3.5	2	2.3		10	3.7	1	1.5		10	4.7	1	1.9	
TNM stage	I	190	54.8	49	56.3	0.444	142	52.0	29	42.6	0.374	93	44.1	26	49.1	0.095
	II	119	34.3	25	28.7		95	34.8	29	42.6		86	40.8	14	26.4	
	IIIa, IIIb, IIIc, IV	38	11.0	13	14.9		36	13.2	10	14.7		32	15.2	13	24.5	
Tumor no.	Single	238	68.6	63	72.4	0.489	190	69.6	39	57.4	0.054	127	60.2	38	71.7	0.122
	Multiple	109	31.4	24	27.6		83	30.4	29	42.6		84	39.8	15	28.3	
Tumor size (cm)	≦5	268	77.2	67	77.0	0.965	204	74.7	50	73.5	0.840	154	73.0	37	69.8	0.644
	>5	79	22.8	20	23.0		69	25.3	18	26.5		57	27.0	16	30.2	
Vascular invasion	No	275	79.3	65	74.7	0.358	206	75.5	54	79.4	0.493	156	73.9	40	75.5	0.819
	Yes	72	20.7	22	25.3		67	24.5	14	20.6		55	26.1	13	24.5	
Portal vein invasion	No	335	96.5	87	100	0.079	265	97.1	66	97.1	0.996	203	96.2	51	96.2	0.995
	Yes	12	3.5	0	0.0		8	2.9	2	2.9		8	3.8	2	3.8	
Surgical procedure	Laparoscopic	69	19.9	15	17.2	0.577	51	18.7	16	23.5	0.368	51	24.2	10	18.9	0.413
	Open surgery	278	80.1	72	82.8		222	81.3	52	76.5		160	75.8	43	81.1	
Postoperative complication	No	310	89.3	78	89.7	0.931	240	87.9	59	86.8	0.797	182	86.3	45	84.9	0.800
	Yes	37	10.7	9	10.3		33	12.1	9	13.2		29	13.7	8	15.1	
Recurrence	No	158	45.5	37	42.5	0.614	115	42.1	23	33.8	0.212	71	33.6	17	32.1	0.828
	Yes	189	54.5	50	57.5		158	57.9	45	66.2		140	66.4	36	67.9	
Postoperative treatment	No	156	45.0	35	40.2	0.427	104	38.1	27	39.7	0.807	67	31.8	19	35.8	0.570
	yes	191	55.0	52	59.8		169	61.9	41	60.3		144	68.2	34	64.2	
Status	Survived	295	85	79	90.8	0.162	165	60.4	37	54.4	0.365	80	37.9	14	26.4	0.118
	Expired	52	15	8	9.2		108	39.6	31	45.6		131	62.1	39	73.6	

**Table 3:** Comparison of predictors for 1-, 3-, and 5-year survival using ANN and LR.

Predictive variables	1-year		3-year		5-year	
	survival		survival		survival	
	ANN	LR	ANN	LR	ANN	LR
Age	*⊚*					
Gender	*⊚*		*⊚*			
Comorbidity	*⊚*		*⊚*		*⊚*	*⊚*
Liver cirrhosis	*⊚*	*⊚*	*⊚*		*⊚*	
*α*-Fetoprotein	*⊚*	*⊚*	*⊚*		*⊚*	
AST		*⊚*				
Total bilirubin	*⊚*		*⊚*			*⊚*
Albumin		*⊚*	*⊚*	*⊚*		
BUN						
Platelet	*⊚*		*⊚*		*⊚*	
ASA classification	*⊚*		*⊚*		*⊚*	
Child-Pugh classification	*⊚*	*⊚*	*⊚*			
TNM stage	*⊚*	*⊚*	*⊚*		*⊚*	
Tumor number				*⊚*	*⊚*	
Tumor size			*⊚*			
Portal vein invasion	*⊚*			*⊚*		*⊚*
Biliary invasion	*⊚*	*⊚*				
Surgical procedure			*⊚*		*⊚*	
Postoperative complication	*⊚*	*⊚*			*⊚*	
Recurrence	*⊚*		*⊚*	*⊚*		*⊚*
Postoperative treatment	*⊚*					

Total	15	8	13	4	9	4

**Table 4:** Comparison of predictive models for 1-, 3-, and 5-year survival using ANN and LR: training data.

	1-year survival		3-year survival		5-year survival	
	(*N* = 347)		(*N* = 273)		(*N* = 211)	
	ANN	LR	ANN	LR	ANN	LR
Accuracy	0.991	0.890	0.985	0.791	0.995	0.801
Sensitivity	0.997	0.986	0.988	0.879	1.000	0.675
Specificity	0.962	0.346	0.981	0.657	0.992	0.878

**Table 5:** Comparison of predictive models for 1-, 3- and 5-year survival using ANN and LR: validation data.

	1-year survival		3-year survival		5-year survival	
	(*N* = 87)		(*N* = 68)		(*N* = 53)	
	ANN	LR	ANN	LR	ANN	LR
Accuracy	0.851	0.885	0.721	0.706	0.792	0.706
Sensitivity	0.886	0.949	0.730	0.757	0.714	0.613
Specificity	0.500	0.250	0.710	0.645	0.821	0.763

## References

[1] http://www.who.int/whosis.

[2] Centers for Disease Control and Prevention (2010). Hepatocellular carcinoma—United States, 2001–2006. *Morbidity and Mortality Weekly Report*.

[3] Capocaccia R, Sant M, Berrino F (2007). Hepatocellular carcinoma: trends of incidence and survival in Europe and the United States at the end of the 20th century. *The American Journal of Gastroenterology*.

[4] Lin XD, Lin LW (2006). Local injection therapy for hepatocellular carcinoma. *Hepatobiliary and Pancreatic Diseases International*.

[5] Hanazaki K, Kajikawa S, Shimozawa N (2000). Survival and recurrence after hepatic resection of 386 consecutive patients with hepatocellular carcinoma. *Journal of the American College of Surgeons*.

[6] Lee KT, Lu YW, Wang SN (2009). The effect of preoperative transarterial chemoembolization of resectable hepatocellular carcinoma on clinical and economic outcomes. *Journal of Surgical Oncology*.

[7] Witten IH, Frank E (2005). *Data Mining: Practical Machine Learning Tools and Techniques*.

[8] Tsai JT, Chou JH, Liu TK (2006). Tuning the structure and parameters of a neural network by using hybrid Taguchi-genetic algorithm. *IEEE Transactions on Neural Networks*.

[9] Das A, Ben-Menachem T, Farooq FT (2008). Artificial neural network as a predictive instrument in patients with acute nonvariceal upper gastrointestinal hemorrhage. *Gastroenterology*.

[10] Ho WH, Chang CS (2011). Genetic-algorithm-based artificial neural network modeling for platelet transfusion requirements on acute myeloblastic leukemia patients. *Expert Systems with Applications*.

[11] Nguyen T, Malley R, Inkelis SH, Kuppermann N (2002). Comparison of prediction models for adverse outcome in pediatric meningococcal disease using artificial neural network and logistic regression analyses. *Journal of Clinical Epidemiology*.

[12] Ho WH, Chou JH, Guo CY (2010). Parameter identification of chaotic systems using improved differential evolution algorithm. *Nonlinear Dynamics*.

[13] Ho WH, Chen JX, Lee IN, Su HC (2011). An ANFIS-based model for predicting adequacy of vancomycin regimen using improved genetic algorithm. *Expert Systems with Applications*.

[14] Tangri N, Ansell D, Naimark D (2008). Predicting technique survival in peritoneal dialysis patients: comparing artificial neural networks and logistic regression. *Nephrology Dialysis Transplantation*.

[15] Peng SY, Peng SK (2008). Predicting adverse outcomes of cardiac surgery with the application of artificial neural networks. *Anaesthesia*.

[16] Luk JM, Lam BY, Lee NPY (2007). Artificial neural networks and decision tree model analysis of liver cancer proteomes. *Biochemical and Biophysical Research Communications*.

[17] Hanai T, Yatabe Y, Nakayama Y (2003). Prognostic models in patients with non-small-cell lung cancer using artificial neural networks in comparison with logistic regression. *Cancer Science*.

[18] Bassi P, Sacco E, De Marco V, Aragona M, Volpe A (2007). Prognostic accuracy of an artificial neural network in patients undergoing radical cystectomy for bladder cancer: a comparison with logistic regression analysis. *BJU International*.

[19] Sargent DJ (2001). Comparison of artificial neural networks with other statistical approaches: results from medical data sets. *Cancer*.

[20] Hall M, Frank E, Holmes G, Pfahringer B, Reutemann P, Witten IH (2009). The WEKA data mining software: an update. *SIGKDD Explorations*.

[21] Yeh CN, Chen MF, Lee WC, Jeng LB (2002). Prognostic factors of hepatic resection for hepatocellular carcinoma with cirrhosis: univariate and multivariate analysis. *Journal of Surgical Oncology*.

[22] Ercolani G, Grazi GL, Ravaioli M (2003). Liver resection for hepatocellular carcinoma on cirrhosis: univariate and multivariate analysis of risk factors for intrahepatic recurrence. *Annals of Surgery*.

[23] Shimozawa N, Hanazaki K (2004). Longterm prognosis after hepatic resection for small hepatocellular carcinoma. *Journal of the American College of Surgeons*.

[24] Liau KH, Ruo L, Shia J (2005). Outcome of partial hepatectomy for large (>10 cm) hepatocellular carcinoma. *Cancer*.

[25] Sasaki A, Iwashita Y, Shibata K, Ohta M, Kitano S, Mori M (2006). Preoperative transcatheter arterial chemoembolization reduces long-term survival rate after hepatic resection for resectable hepatocellular carcinoma. *European Journal of Surgical Oncology*.

[26] Robinson CJ, Swift S, Johnson DD, Almeida JS (2008). Prediction of pelvic organ prolapse using an artificial neural network. *American Journal of Obstetrics and Gynecology*.

[27] Royston P, Altman DG (2010). Visualizing and assessing discrimination in the logistic regression model. *Statistics in Medicine*.

[28] Altman DG, Vergouwe Y, Royston P, Moons KGM (2009). Prognosis and prognostic research: validating a prognostic model. *BMJ*.

[29] Lo A, Chiu YY, Rødland EA, Lyu PC, Sung TY, Hsu WL (2009). Predicting helix-helix interactions from residue contacts in membrane proteins. *Bioinformatics*.

[30] Tilmanne J, Urbain J, Kothare MV, Wouwer AV, Kothare SV (2009). Algorithms for sleep-wake identification using actigraphy: a comparative study and new results. *Journal of Sleep Research*.

[31] Green M, Björk J, Forberg J, Ekelund U, Edenbrandt L, Ohlsson M (2006). Comparison between neural networks and multiple logistic regression to predict acute coronary syndrome in the emergency room. *Artificial Intelligence in Medicine*.

[32] Lin SP, Lee CH, Lu YS, Hsu LN (2006). A comparison of MICU survival prediction using the logistic regression model and artificial neural network model. *The Journal of Nursing Research*.

[33] Sakai S, Kobayashi K, Toyabe SI, Mandai N, Kanda T, Akazawa K (2007). Comparison of the levels of accuracy of an artificial neural network model and a logistic regression model for the diagnosis of acute appendicitis. *Journal of Medical Systems*.

[34] Wang CH, Mo LR, Lin RC, Kuo JJ, Chang KK, Wu JJ (2008). Artificial neural network model is superior to logistic regression model in predicting treatment outcomes of interferon-based combination therapy in patients with chronic hepatitis C. *Intervirology*.

[35] Hannula M, Huttunen K, Koskelo J, Laitinen T, Leino T (2008). Comparison between artificial neural network and multilinear regression models in an evaluation of cognitive workload in a flight simulator. *Computers in Biology and Medicine*.

[36] Alkan A, Koklukaya E, Subasi A (2005). Automatic seizure detection in EEG using logistic regression and artificial neural network. *Journal of Neuroscience Methods*.

[37] Eftekhar B, Mohammad K, Ardebili HE, Ghodsi M, Ketabchi E (2005). Comparison of artificial neural network and logistic regression models for prediction of mortality in head trauma based on initial clinical data. *BMC Medical Informatics and Decision Making*.

[38] Das A, Ben-Menachem T, Farooq FT (2008). Artificial neural network as a predictive instrument in patients with acute nonvariceal upper gastrointestinal hemorrhage. *Gastroenterology*.

[39] Trtica-Majnaric L, Zekic-Susac M, Sarlija N, Vitale B (2010). Prediction of influenza vaccination outcome by neural networks and logistic regression. *Journal of Biomedical Informatics*.

[40] Simon D, Boring JR (1990). *Clinical Methods: The History, Physical, and Laboratory Examinations*.

